# Biomineralized *in situ* catalytic nanoreactor integrated microneedle patch for on demand immunomodulator supply to combat psoriasis

**DOI:** 10.7150/thno.101845

**Published:** 2024-10-07

**Authors:** Xiaodie Li, Minglong Chen, Xinni He, Jinghang Cong, Wanchen Zhao, Yanping Fu, Chao Lu, Chuanbin Wu, Xin Pan, Guilan Quan

**Affiliations:** 1 State Key Laboratory of Bioactive Molecules and Druggability Assessment, Jinan University, Guangzhou 511443, China.; 2 College of Pharmacy, Jinan University, Guangzhou 511443, China.; 3 Department of Polymer Science and Engineering, University of Science and Technology of China, Hefei 230026, China.; 4 Key Laboratory of Precision and Intelligent Chemistry, University of Science and Technology of China, Hefei 230026, China.; 5 School of Pharmaceutical Sciences, Sun Yat-sen University, Guangzhou 510006, China.

**Keywords:** adenosine, immunoregulation, *in situ* catalytic nanoreactor, microneedles, psoriasis

## Abstract

The endogenous immunomodulator adenosine (ADO) was expected to be potentialized as an efficacious mediator to combat psoriasis. However, its efficacy is severely hindered by its poor metabolic stability and insufficient accumulation at the dermatological lesions.

**Methods:** In this study, a biomineralized *in situ* catalytic nanoreactor was delicately customized by encapsulating ADO precursor (adenosine monophosphate, AMP) within the internal porous skeleton of zeolitic imidazolate framework-90, followed by the biomineralization of the AMP catabolic enzyme on the outer layer. The nanocrystals were then incorporated into a dissolving microneedles patch, which was designed to deliver drugs with precision into the cutaneous lesion and enhance the efficacy of psoriasis treatment.

**Results:** Upon penetration into the skin, the nanoreactors were released and underwent a gradual collapse of their structure, releasing AMP when exposed to the acidic microenvironment. Meanwhile, the acidic pH could trigger an *in situ* catalytic reaction to continuously produce ADO. This system yielded remarkable results in a psoriasis-like mouse model. The mechanism study demonstrated that this system could substantially reshape the inflammatory ecosystem by inhibiting the keratinocyte hyperplasia, reducing inflammatory cytokine expression, and regulating the infiltration of immune cells.

**Conclusion:** The *in situ* catalytic nanoreactor integrated microneedle patch is a promising modular platform for co-delivery of prodrugs and their catabolic enzymes, offering a potential solution for various diseases.

## Introduction

Psoriasis is a prevalent dermatological autoimmune disorder, clinically manifested by pruritus, hyperkeratotic plaques, erythema, and dermal inflammatory infiltration 1, 2. With a high prevalence and incidence, psoriasis affects more than 125 million people worldwide and has been classified as a serious non-communicable disease by the World Health Organization 3, 4. Although the precise mechanism underlying psoriasis pathogenesis remains unclear, there is a consensus that excessive activation of the adaptive immune system plays a pivotal role in its development 5, 6. Once psoriasis has manifested, a series of cells, including plasmacytoid dendritic cells (DCs) and macrophages, are excessively activated to promote the secretion of cytokines, including tumor necrosis factor-alpha (TNF-α), interleukin-6 (IL-6), interleukin-17 (IL-17), interleukin-22 (IL-22), and interleukin-23 (IL-23) 1, 7. These cytokines play a pivotal role in orchestrating the proliferation and differentiation of related immune cells, which in turn induces an inflammatory cascade reaction and leads to the hyperproliferation of keratinocytes and massive infiltration of immune cells into lesional sites.

The clinical treatment of psoriasis remains a significant challenge due to its chronic nature and high recurrence rate 8. It is conceivable that conventional therapies such as corticosteroids, methotrexate, and cyclosporine may cause cumulative toxicity when administered over an extended period of time. The introduction of biologics has constituted a significant advance in the treatment of psoriasis over the past two decades 9-11. These agents, including TNF-α inhibitors, IL-17 antagonists, and IL-12/23 antagonists 12, have been demonstrated to effectively ameliorate the symptoms of psoriasis. This is achieved by alleviating pruritus, erythema, scales, and other uncomfortable conditions, through the regulation of cytokine function within the immune system and the inhibition of inflammatory response 13. Despite their outstanding therapeutic efficacy, these therapeutic biologics continue to exhibit certain drawbacks, including the potential for serious immune suppression, the risk of opportunistic infection, and the high cost of treatment 14. Adenosine (ADO) is an endogenous immunosuppressive agent that can regulate the immune system and maintain homeostasis by affecting the metabolism of specific immune cells 15-17. As a purine nucleoside, ADO mediates anti-inflammatory effects through type 1 purinergic (P1) receptors, including A1R, A2AR, A2BR, and A3R. In the context of the adaptive immune system, ADO has the capacity to modulate the activity and proliferation of CD4^+^ T cells, while simultaneously reinforcing the function of immunosuppressive cells through binding to receptors A2AR and A2BR 16. This results in the restrained secretion of pro-inflammatory cytokines. It was therefore anticipated that ADO would act as a highly efficacious mediator in the treatment of psoriasis. Currently, two brands of ADO injection, Adenocard^@^ and Adenoscan^@^, have been approved by the US Food and Drug Administration for antiarrhythmic and myocardial perfusion scintigraphy, respectively 18, 19. For the treatment of psoriasis, the systemic administration of the drug is prone to result in insufficient drug accumulation in the cutaneous lesions, which in turn leads to a reduction in the drug's bioavailability and the occurrence of toxic side effects. Moreover, the efficacy of ADO is severely limited due to its poor metabolic stability (circulating half-life <10 s) 20. Therefore, it is extremely necessary to develop a topical delivery system for the continuous supply of ADO in the management of psoriasis.

Recently, researchers have been endeavoring to identify novel adenosine derivatives with enhanced potency and potential applications 21, 22. Adenosine monophosphate (AMP), a prodrug of ADO, can be hydrolyzed into ADO through the catalysis of acid phosphatase (ACP) by phosphatase reaction under acidic conditions 23, 24. Psoriasis is a dermatological disorder with inflammatory characteristics that exhibits a typical acidic microenvironment. This can be attributed to various factors, including metabolic imbalance and the excretion of excess sweat and sebum 25. Thus, we postulated that AMP could be explored as a potential candidate for an efficacious psoriasis treatment under the catalysis of ACP.

**Scheme 1:**
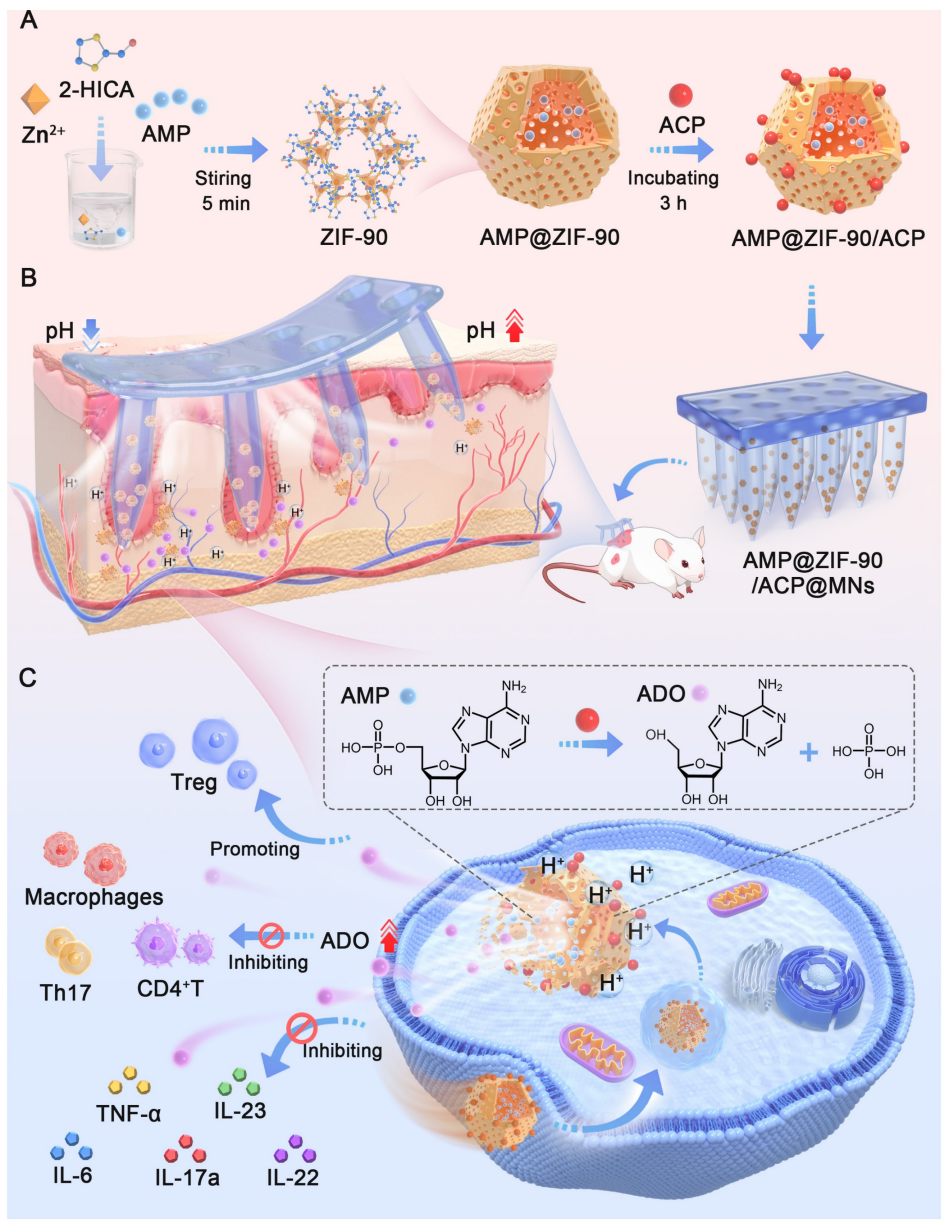
Schematic diagram of AMP@ZIF-90/ACP integrated microneedle patch for on-demand ADO supply to combat psoriasis.

In this study, hydrophilic metal-organic frameworks (MOFs), zeolitic imidazolate framework-90 (ZIF-90) crystals were meticulously customized to encapsulate AMP within their internal skeleton. The AMP catabolic enzyme ACP was biomimetically mineralized on the outer layer. ZIF-90 was selected for its porous structure, which allows for guest loading, catalysis, energy storage, and separation 26-28. Additionally, ZIF-90 exhibits pH-responsiveness, whereby its structure gradually collapsed under acidic conditions 29. The resulting AMP@ZIF-90/ACP nanocrystals were then incorporated into a dissolving microneedle (MNs) patch. MNs are equipped with hundreds of micrometer-sized needles that can directionally pierce through the skin* stratum corneum* barrier in a minimally invasive manner, generating an array of microchannels that facilitate precise “zone accumulation” at the cutaneous lesion sites 30-33. The low drug loading capacity and uncontrollable release profile of conventional MNs have impeded their application. The integration of nanotechnology can address these issues by enabling the loading of various drugs and facilitating controlled release. Upon penetration into the skin, the AMP@ZIF-90/ACP was released, accompanied by the dissolution of the needle tips, and achieved a three-dimensional distribution at the deep lesion tissue. Subsequently, the compact structure of ZIF-90 underwent a gradual collapse, releasing AMP in response to the acidic dermal microenvironment characteristic of psoriasis. Meanwhile, the acidic pH could initiate an *in situ* catalytic reaction, resulting in the continuous generation of ADO and the subsequent exertion of its immunosuppressive effect (**Scheme 1**). Following administration in a psoriasis-like mouse model, AMP@ZIF-90/ACP@MNs exhibited a favorable immunomodulatory effect, reducing the infiltration of inflammatory macrophages, DCs, and T cells into the skin and decreasing the expression of inflammatory cytokines, including TNF-α, IL-6, IL-17A, and IL-23. It was therefore anticipated that this system would provide a modular platform for the co-delivery of prodrugs and their catabolic enzymes to treat a wide range of diseases.

## Materials and methods

### Materials

Zinc nitrate hexahydrate [Zn(NO_3_)_2_·6H_2_O] was obtained from Aladdin Biochemical Technology Co., Ltd. (Shanghai, China). Imidazole-2 carboxcaldehyde, AMP, and ACP were purchased from Macklin Biochemical Technology Co., Ltd. (Shanghai, China). ADO was supplied by Sigma Aldrich Trading Co., Ltd. (St. Louis, USA). Sodium hyaluronic acid (HA) was obtained from Bloomage Freda Biopharm Co., Ltd. (Shanxi, China). Polyvinyl pyrrolidone (PVP) K90 was kindly donated by MB CHEM Co., Ltd. (Bangkok, Thailand). PVP K30 and diamidino-2-phenylindole (DAPI) were acquired from Beijing Solarbio Science & Technology Co., Ltd. (Beijing, China). CCK-8 and ELISA kits were purchased from Biosharp Technology Co., Ltd. (Beijing, China). Fluorescent labeled antibodies including anti-45 (Lot: 557659), anti-CD11c (Lot: 558079), anti-CD3 (Lot: 553692), anti-CD4 (Lot: 563106), anti-FoxP3(Lot: 563101), anti-80 (Lot: 560016), anti-CD86 (Lot: 553692), and anti-CD25 (Lot: 557192) were obtained from Becton Dickinson Medical Devices Co., Ltd. (Shanghai, China). All reagents were used as received without further purification.

### Cells and animals

HaCaT cells (human immortalized epidermal cells) and Raw 264.7 cells (mouse mononuclear macrophage leukemia cells) were cultured with the aseptic DMEM medium at 37 °C under a 5% CO_2_ atmosphere. BALB/c mice were purchased from the Guangdong Medical Laboratory Animal Center (Guangzhou, China) and all animal experiments were approved by the Laboratory Animal Welfare and Ethics Committee of Jinan University with the National Institute of Health and Nutrition Guidelines for the care and use of laboratory animals (Application No: 103509; Approval No: IACUC-20240327-05).

### Synthesis and characterization of biomineralized ZIF-90

The ZIF-90 was synthesized referred to a previous study with minor modifications 34. Briefly, 7.5 mg of PVP K30 and 72 mg of imidazole-2-carboxaldehyde were added into a 10 mL glass beaker containing 2.5 mL of deionized water. The resulting mixture was then stirred continuously for 10 min at 80°C until a transparent solution was obtained (Solution A). Subsequently, 46 mg of Zn(NO_3_)_2_·6H_2_O was dissolved in 250 μL of deionized water as solution B, which was added dropwise into solution A and continued to be stirred at 300 rpm for 5 min. The resulting raw product was collected by centrifugation at 15,000 rpm for 10 min, washed with deionized water three times to remove any residual reagents, and dispersed in 1 mL of deionized water for further use.

AMP@ZIF-90 was synthesized through a facile one-pot method. Briefly, 10 mg of AMP was dissolved in 1 mL of deionized water and mixed with solution A, followed by the addition of solution B. The remaining procedures were consistent with those previously described. Then, the ACP solution was added to the resulting AMP@ZIF-90 suspension and incubated under static conditions at room temperature for 3 h. Finally, after being washed with deionized water, the collected AMP@ZIF-90/ACP nanocrystals were dispersed in 1 mL of deionized water.

The morphology of ZIF-90, AMP@ZIF-90, and AMP@ZIF-90/ACP, as well as the energy dispersive X-ray (EDX) analysis of AMP@ZIF-90/ACP were investigated by scanning electron microscope (SEM, TESCAN MIRA LMS, Delong Instruments Ltd., USA). The surface zeta potential of the various samples was measured by dynamic light scattering (DLS, Zetasizer Nano ZS90, Malvern Instruments Ltd., UK). The ultraviolet-visible (UV-Vis) absorption spectra of the various samples were obtained using a spectrophotometer (UV-Vis, V-730, Jasco Instruments Ltd., Italy), and the crystalline structure of the raw materials and synthetic products was characterized using a powder X-ray diffraction (pXRD) apparatus (Miniflex600, Rigaku Instruments Ltd., Japan).

The obtained AMP@ZIF-90/ACP was subjected to high-performance liquid chromatography (HPLC, Agilent 1200, Agilent Instruments Ltd., Germany) and Bradford Method protein assay kit analysis to determine the concentration of AMP and ACP, respectively. The encapsulation efficiency (EE) and drug loading (DL) were calculated according to the following formulas:

EE (%) = [weight of drug in nanocrystals /weight of total added drug] × 100%

DL (%) = [weight of drug in nanocrystals /weight of total nanocrystals] × 100%

### pH-responsive behavior of AMP@ZIF-90/ACP

To investigate the pH-responsive degradation behavior, an AMP@ZIF-90/ACP suspension was immersed in pH 5.5, 6.5, 7.4, and 8.5 PBS, respectively, under stirring at 500 rpm at 37 ℃. Aliquot samples (1.5 mL) were withdrawn at predetermined time points, and filtered through a 0.22 μm Millipore filter. The samples were then analyzed by HPLC to determine the ADO and AMP content. An equal volume of fresh medium was added to compensate for the loss due to sampling. Additionally, the nanocrystals were collected for morphological examination via SEM at designated time points.

### Cytotoxicity assessment and cellular uptake analysis

The HaCaT cells and Raw264.7 cells were cultured in 96-well plates at 37 °C under a 5% CO_2_ atmosphere overnight. Subsequently, the original medium was replaced with 100 μL of fresh medium containing varying concentrations of ZIF-90, AMP@ZIF-90, and AMP@ZIF-90/ACP. After incubation for 24 h, the CCK8 solution was added and allowed to incubate for another 1 h. Thereafter, the optical density (OD) value of each well was measured at 450 nm using an automatic enzyme-linked immunosorbent assay system (Spark10M, Tecan Instruments Ltd., Switzerland).

To evaluate the cellular uptake behavior, coumarin 6 (C6) was used as a labeling agent for the nanocrystal. Briefly, the C6@ZIF-90 suspension was co-incubated with HaCaT cells and Raw264.7 cells for a specified period (0.5, 2, 4 h), respectively. Subsequently, the cells were fixed with 4% polyformaldehyde for 15 min in the dark, stained with DAPI for 10 min, and visualized by CLSM (LSM 900, Zeiss Instruments Ltd., Germany). To quantify the cellular uptake, the nanocrystal-treated cells were harvested and resuspended in PBS. The collected cells were then analyzed by flow cytometry (FACS Canto II, Becton Dickinson Medical Devices Ltd., USA). The mean fluorescence intensity (MFI) was calculated using FlowJo software.

### Fabrication and characterization of AMP@ZIF-90/ACP@MNs

The nanocrystal-encapsulated MNs patch was fabricated via a modified centrifugation method according to our previous report 35. Firstly, the obtained AMP@ZIF-90/ACP suspension was blended with 350 mg of HA powder to form a needle matrix solution. Subsequently, the needle matrix solution was deposited into the microchannel of the female mold under centrifugation at 4000 rpm for 5 min at 20 °C. Once the remaining solution outside of the microcavities had been removed, the female mold was subjected to another 30 min of centrifugation and subsequently dried in a desiccator for 24 h at room temperature. Afterward, an equal volume of the needle matrix solution was introduced into the female mold for a second round of centrifugation under identical conditions. Next, a 40% PVP K90 ethanol solution (w/v) was added to form the base part after centrifugation. Ultimately, the AMP@ZIF-90/ACP@MNs patch was carefully removed from the female mold after a two-day drying period.

An electron microscope, SEM, and digital camera were employed to evaluate the morphology of the MNs. The distribution of nanocrystals encapsulated within the MNs was observed by CLSM.

### *In vitro* skin penetration test

To evaluate the capacity for skin penetration, a piece of rhodamine B@ZIF-90@MNs was pressed into the excised mouse skin for 2 min. Following the removal of the MN base, the penetration site was imaged using a digital camera. The longitudinal dimension of the generated microchannel was investigated through H&E staining.

### Dissolution and diffusion behavior testing

The dissolution and diffusion behavior of the MN patch was evaluated using a skin-mimicking agarose gel. Briefly, a piece of AMP@ZIF-90/ACP@MNs was inserted into the agarose gel. Subsequently, the samples were observed under an upright microscope. The dissolution and diffusion of the needles within the gel were recorded until the needle tips were completely separated from the base part.

### *In vivo* biodistribution study

To investigate the *in vivo* biodistribution, imiquimod cream was applied to the shaved dorsal skin of a mouse with a size of 2 cm × 3 cm for 6 consecutive days, thereby establishing a psoriasis-like model. Subsequently, the eight mice exhibiting psoriasis-like symptoms were randomly assigned to two groups: MNs group and the subcutaneous injection (SC) group. In the MNs group, a piece of rhodamine B@ZIF-90@MNs was pressed to the dorsal psoriatic skin with the thumb for 2 min, after which it was fixed in place with medical tape for 30 min. In the SC group, the rhodamine B@ZIF-90 suspension was administered via an insulin syringe. The fluorescence signals of the mice were detected at 2, 4, 8, 12, and 24 h, respectively, using the IVIS (IVIS Lumina Series III, PerkinElmer Instruments Ltd., USA). Furthermore, the mice were euthanized at 24 h, and the major organs (heart, liver, spleen, lung, kidney, and lymph) were dissected to observe the distribution of the fluorescence signal.

### *In vivo* anti-psoriasis efficacy study

The* in vivo* anti-psoriasis study was conducted using specific pathogen-free (SPF) BALB/c male mice (7-8 week age). A psoriasis-like mouse model was established by the application of imiquimod cream to the mouse skin for 6 consecutive days. The mice were randomly divided into five groups: two comparison groups with normal group (G1, Normal) and psoriasis-like model group (G2, Control), and three treatment groups (G3, HA@MNs; G4, ADO@ZIF-90@MNs; G5, AMP@ZIF-90/ACP@MNs). For the purpose of treatment, the corresponding MN patches were applied to the lesion skin at two-day intervals, commencing on the second day following the administration of imiquimod and continuing until the seventh day after the third doses were administered. During the course of treatment, the PASI scores (ranging from 0 to 4) for desquamation, erythema, and skin thickness were evaluated on a daily basis, according to the severity of each symptom. On Day 7, the body weight and spleen weight were recorded to calculate the spleen index, defined as the ratio of spleen spleen weight (mg) to body weight (g). Following euthanasia, the skin lesions of different groups were acquired for H&E staining, allowing for the measurement of epidermis thickness.

### Evaluation of the immunosuppressive effect

On Day 7, psoriasis-like skin lesions and draining lymph nodes were dissected for analysis of immune cell phenotyping using flow cytometry. Briefly, a single-cell suspension of psoriasis-like skin lesions was prepared and stained with fluorescence-labeled antibodies, including anti-45-APC-CY7, anti-CD11c-PE-CY7, anti-CD3-FITC, anti-CD4-BV510, and anti-FoxP3-PE. Additionally, a single-cell suspension of lymph nodes was also prepared and stained with fluorescence-labeled antibodies, including anti-45-APC-CY7, anti-CD11c-PE-CY7, anti-80-APC, anti-CD86-PE, anti-CD3-FITC, anti-CD4-BV510, anti-CD25-APC, and anti-FoxP3-PE. The concentration of relevant cytokines (TNF-α, IL-6, IL-17A, and IL-23) in serum were quantified using ELISA kits.

### *In vivo* inflammatory response and histological analysis

To evaluate the inflammatory response, whole blood samples from the control and AMP@ZIF-90/ACP@MNs groups were collected 12 h after the final administration for biomarker analysis. Following euthanasia, psoriasis-like skin lesions were dissected for immunofluorescence staining of CD3, CD68, IL-6, IL-17A, IL-22, and Ki67, respectively.

### *In vivo* safety assessment

To assess the skin recovery process, a piece of AMP@ZIF-90/ACP@MNs was inserted into the dorsal skin of a mouse for 2 min. Following the removal of the base portion, the insertion site was examined via H&E staining at 0, 5, 10, 15, and 20 min following the administration of the MNs patch. The body weight of the mice was recorded on a daily basis throughout the course of the treatment period. At Day 7, a histological examination of the major organs (heart, liver, spleen, lung, and kidney) of the various groups was conducted using H&E staining.

### Statistical analysis

All the data are presented as mean ± standard deviation (SD). The statistical analysis was conducted using a one-way analysis of variance, followed by Tukey's post-test. A *P* value of less than 0.05 was considered statistically significant (*P* < 0.05, **P < 0.01, ***P < 0.001, and ****P < 0.0001).

## Results and Discussion

### Synthesis and characterization of AMP@ZIF-90/ACP

**Figure 1:**
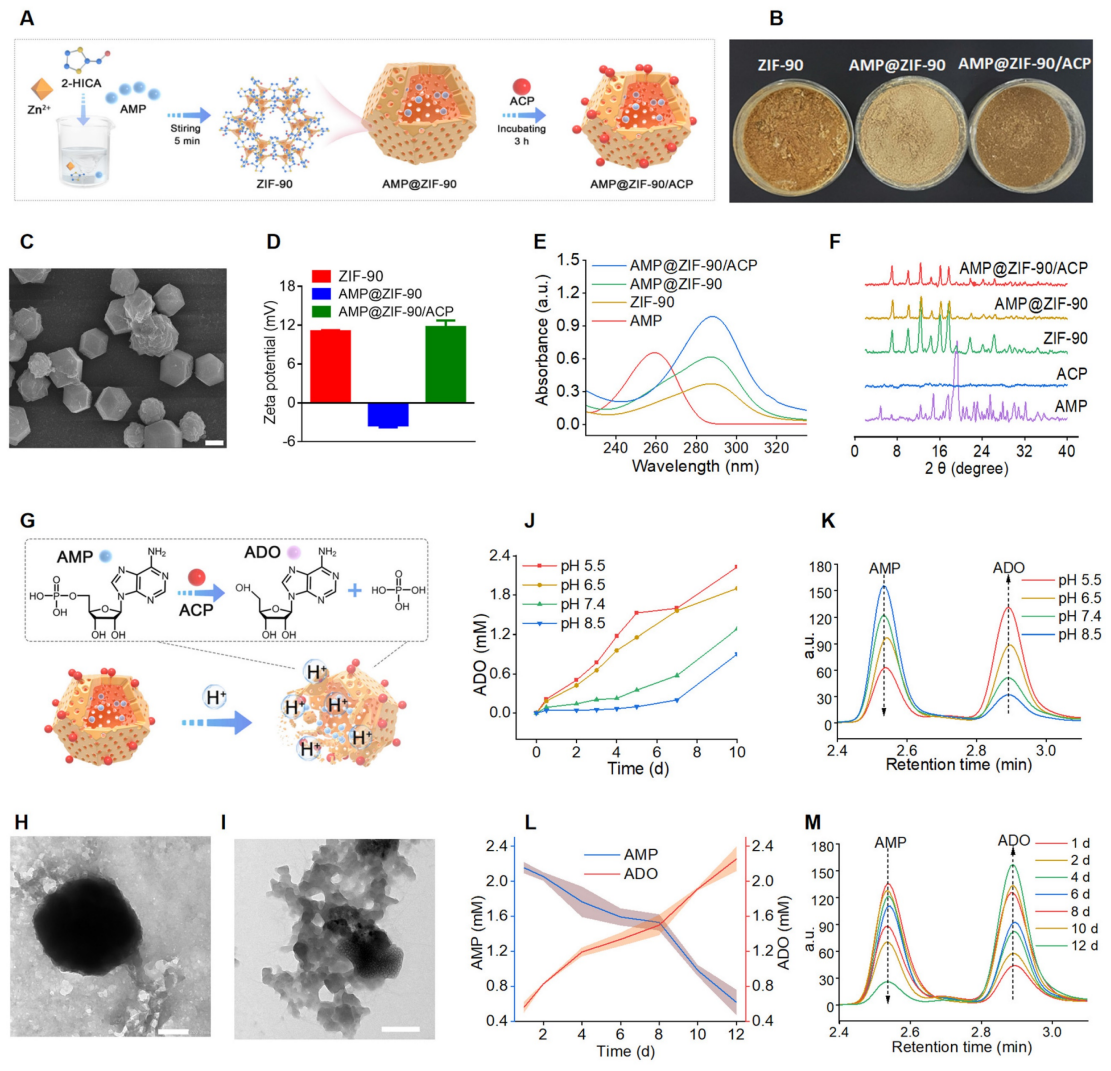
Synthesis and characterization of AMP@ZIF-90/ACP. (A) Schematic illustration of preparation procedures. (B) Digital photograph of the three products. (C) SEM image of ZIF-90. Scale bar: 4 μm. (D) Zeta potential of ZIF-90, AMP@ZIF-90, and AMP@ZIF-90/ACP. (E) UV-Vis absorption spectra of various samples. (F) pXRD spectra of the raw materials and synthetic products. (G) Schematic diagram of pH-responsive degradation and reaction of AMP@ZIF-90/ACP in the acidic medium. (H) TEM image of ZIF-90 and (I) AMP@ZIF-90/ACP after immersion in PBS (pH 5.5) for 48 h. Scale bar: 200 nm. (J) The ADO concentration and (K) corresponding chromatograms after immersing AMP@ZIF-90/ACP at different pH conditions. (L) The variations of time-dependent AMP and ADO concentrations, and (M) corresponding chromatogramsafter immersing AMP@ZIF-90/ACP in PBS (pH 5.5).

The synthesis procedure of the biomineralized nanoreactor AMP@ZIF-90/ACP is illustrated in **Figure 1A**. It has been demonstrated that the structure and activity of proteins may be altered following their adsorption into diverse MOFs. ZIF-8, which exhibits excellent biocompatibility and a high specific surface area, has been employed widely as a drug carrier 36, 37. However, a previous study demonstrated that the enzyme activity of ACP was diminished following its encapsulation in ZIF-8. This phenomenon could be attributed to the conformational alteration of ACP, which resulted from the robust hydrophobic interaction between the protein and ZIF-8 38. Therefore, ZIF-90, constructed by imidazole-2 carboxaldehyde with a hydrophilic aldehyde group, was explored as a potential carrier for AMP and ACP. Compared to ZIF-8 (**Figure S1**), the enzymatic activity of ACP was effectively retained after loading in ZIF-90.

AMP was directly incorporated during the synthesis of the ZIF-90 skeleton by forming weak coordination bonds between the phosphate groups of AMP and Zn^2+^ ions in aqueous media, which proved advantageous for achieving a high loading capacity with uniform distribution throughout the framework matrix. Subsequently, the mineralization of ACP was conducted under weakly alkaline conditions (pH 8.5), given the sensitivity of AMP@ZIF-90 to acidic media. The distinctive colors of the nanoparticles at varying synthesis stages are illustrated in **Figure 1B**. As shown in **Figure 1C**, the synthesis of a rhombic dodecahedron-shaped ZIF-90 with a smooth surface was successfully completed. Following the loading of AMP into ZIF-90, a change in color was observed, with the nanoparticles turning from claybank (ZIF-90) to off-white (AMP@ZIF-90). The diameter of the nanoparticles was approximately 50 nm (**Figure S2A**). The final product, AMP@ZIF-90/ACP, exhibited a tawny coloration and a slightly larger particle size of 80 nm (**Figure S2B**). As illustrated in **Figure 1D**, the ZIF-90 exhibited a positive charge of 11.1 eV, which can be attributed to the presence of abundant imidazole rings. In contrast, the zeta potential of AMP@ZIF-90 decreased to -3.5 eV due to the incorporation of phosphate groups from AMP. Following the mineralization of the positively charged ACP, the zeta potential of AMP@ZIF-90/ACP was observed to undergo a reversal, reaching a value of -11.7 eV. Moreover, to ascertain the composition of the nanocrystals, the elemental composition was validated through energy dispersive X-ray (EDX) analysis (**Figure S3**). The results demonstrated the uniform distribution of elements throughout the nanoparticles, including C, N, O, P, which originated from both AMP and ACP, S, which was specifically present in ACP, and Zn. Altogether, these results demonstrated that AMP was successfully loaded into the internal skeleton of ZIF-90, and ACP was mineralized on its outer layer. The encapsulation efficiency of AMP was calculated to be 97.3%, and its drug loading efficiency was 18.2%. Similarly, the encapsulation efficiency of ACP was calculated to be 74.8%, and its drug loading efficiency was 8.6%.

In addition, the UV-Vis absorption spectra were recorded and are illustrated in **Figure 1E**. Free AMP exhibited a characteristic absorption peak at 259 nm, while bare ZIF-90 exhibited a maximum absorption peak at 288 nm. Following the loading of AMP, both AMP@ZIF-90 and AMP@ZIF-90/ACP exhibite the typical peaks at 288 nm, which are likely attributed to the interaction between AMP and ZIF-90. The crystalline structure of the samples was further elucidated through pXRD analysis. As illustrated in **Figure 1F**, the diffraction peaks of AMP@ZIF-90 and AMP@ZIF-90/ACP remained consistent with those of ZIF-90, exhibiting no characteristic peaks of free AMP. The results demonstrated that the incorporation of AMP and the mineralization of ACP did not alter the crystalline structure of ZIF-90. Instead, AMP was incorporated in an amorphous or molecular state.

### pH-responsiveness of AMP@ZIF-90/ACP

ZIF-90 is a pH-responsive biodegradable carrier that exhibits remarkable stability in physiological conditions. However, it undergoes gradual degradation in acidic environments due to the acid-base reaction between imidazole groups and hydrogen ions 39, which is advantageous for controlled drug delivery (**Figure 1G**). The morphology of ZIF-90 and AMP@ZIF-90/ACP was investigated following a 48 h incubation period in PBS (pH 5.5). As illustrated in **Figure 1H- I**, the nanocrystals exhibited structural distortion, including collapsed frameworks and indistinct degradation fragments. Given that the Since the pH of the skin and psoriasis-like skin lesions typicall ranges between 5.2 and 5.9 40, 41, the encapsulated AMP can be released gradually, accompanied by the collapse of the ZIF-90 structure. It has been demonstrated that the enzyme ACP exhibits the highest catalytic activity under acidic conditions, with a pH range of 5 to 6 24. The ADO generated by the ACP-based catalytic reaction was assayed at varying pH values. As presented in **Figure 1J-K,** the hydrolysis rate of ACP in an acidic condition (pH < 7) was markedly higher than that observed in a neutral or alkaline environment (pH > 7). Furthermore, given the acidic nature of the phosphoric acid, it can be postulated that the phosphoric acid produced during the reaction should simultaneously accelerate the *in situ* catalytic reaction. Moreover, ACP was observed to hydrolyze AMP into ADO in a time- and concentration-dependent manner (**Figure 1L-M, Figure S4**). These results indicate that the constructed system has the potential to serve as a convenient and effective *in situ* catalytic nanoreactor. It can be activated in response to an acidic pathological microenvironment, thereby continuously supplying ADO for immunoregulation against psoriasis.

### *In vitro* cytotoxicity and cellular uptake behavior

The *in vitro* cytotoxicity against HaCaT cells and Raw264.7 cells was evaluated using a CCK-8 assay. As displayed in **Figure S5**, both the bare ZIF-90 and the drug-loaded ZIF-90 exhibited no discerible cytotoxicity across the entire concentration range, thereby indicating the favorable biocompatibility of the developed systems. This finding is consistent with the results of a previous study, in which AMP was identified as an energy storage and transfer molecule that is beneficial for cell growth^42^.

To ascertain the cellular uptake behavior of nanocrystals, the C6-labeled samples C6@ZIF-90 were respectively incubated with HaCaT cells and Raw264.7 cells for 4 h. Following this incubation period, the samples were visualized using a confocal laser scanning microscope (CLSM), and a flow cytometric analysis was conducted. As illustrated in **Figure S6A**, the green fluorescence indicative of C6@ZIF-90 in both cell types exhibited a gradual increase over time, thereby demonstrating a time-dependent cellular uptake behavior. The flow cytometric analysis demonstrated that the uptake by Raw264.7 cells had nearly reached saturation after an incubation period of 2 h (**Figure S6B**).

### Fabrication and characterization of AMP@ZIF-90/ACP@MNs

As a novel transdermal drug delivery system, MNs have been employed extensively over recent decades to encapsulate a range of substances, including small molecules, biomacromolecules, and nanoparticles, for the treatment of diverse diseases 43. MNs arrays are capable of delivering the therapeutic agents directly into the dermal tissue through the formation of microchannels, which enables the successful penetration of the *stratum corneum* barrier. This approach offers an improved therapeutic efficacy compared to traditional methods. It is nnoteworthy that MNs with customized lengths can be administered in a minimally invasive mannerm, avoiding contact with sensory nerve endings and blood capillaries. It is therefore anticipated that transdermal drug delivery via MN patches will emerge as a highly attractive avenue for the treatment of dermatological disorders.

**Figure 2:**
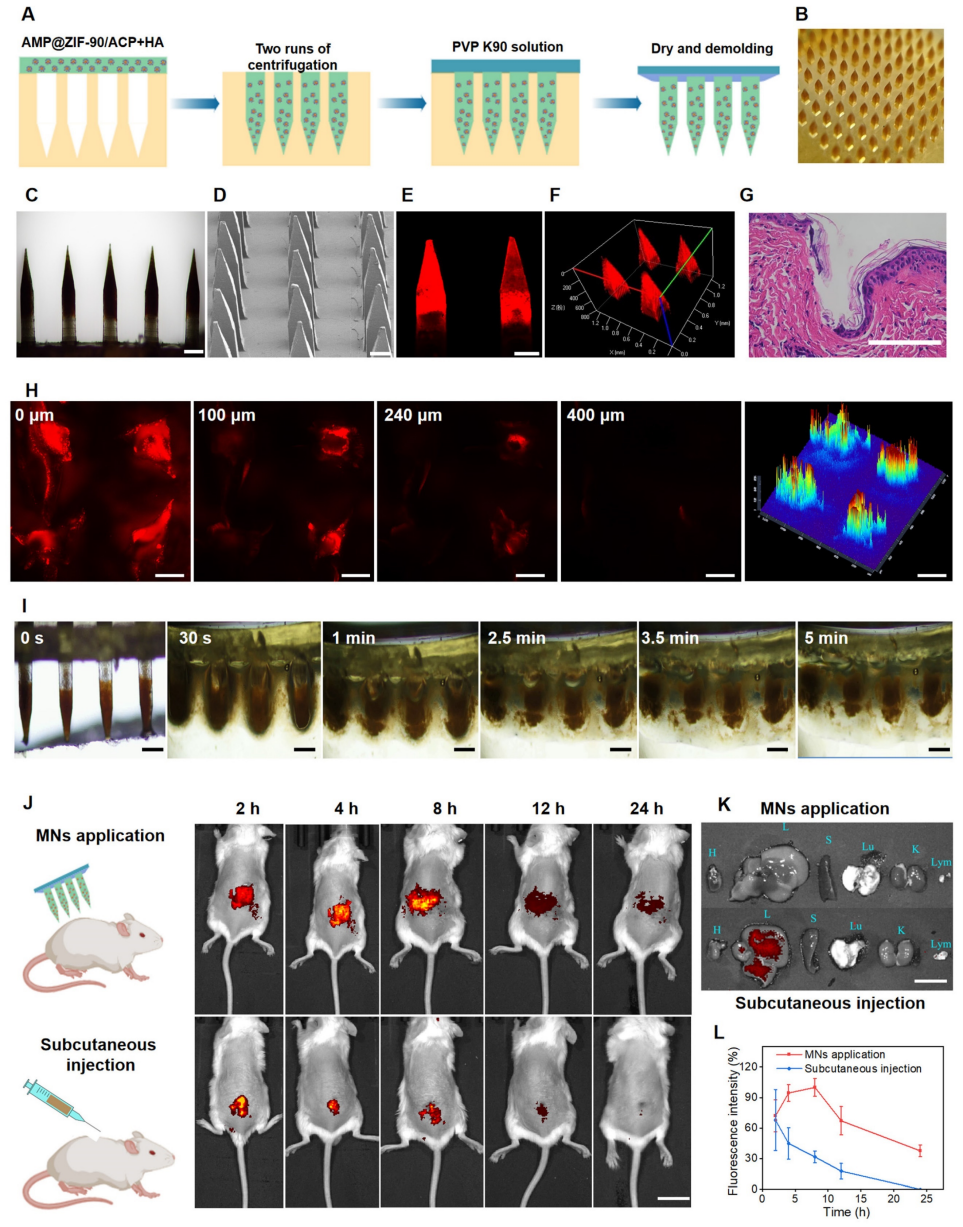
Fabrication and characterization of AMP@ZIF-90/ACP@MNs. (A) Schematic diagram of preparation procedures. (B) Digital photograph, (C) Electron microscope image, and (D) SEM image of AMP@ZIF-90/ACP@MNs. (E) 2D CLSM micrograph and (F) 3D reconstruction image of C6@ZIF-90@MNs. (G) H&E staining section of inserted mice dorsal skin. (H) CLSM tomography of C6 across the mice skin after MNs administration. (I) The dissolution behavior of AMP@ZIF-90/ACP@MN. (J) Fluorescent images of psoriasis-like model mice after administration of C6@ZIF-90@MNs and subcutaneous injection of C6@ZIF-90. (K) Fluorescent image of major organs 24 h post-administration. (L) Total fluorescent intensity of psoriasis-like model mice at different time intervals after administration of C6@ZIF-90@MNs and subcutaneous injection of C6@ZIF-90 (*n* = 3). All scale bars: 200 μm.

**Figure 3:**
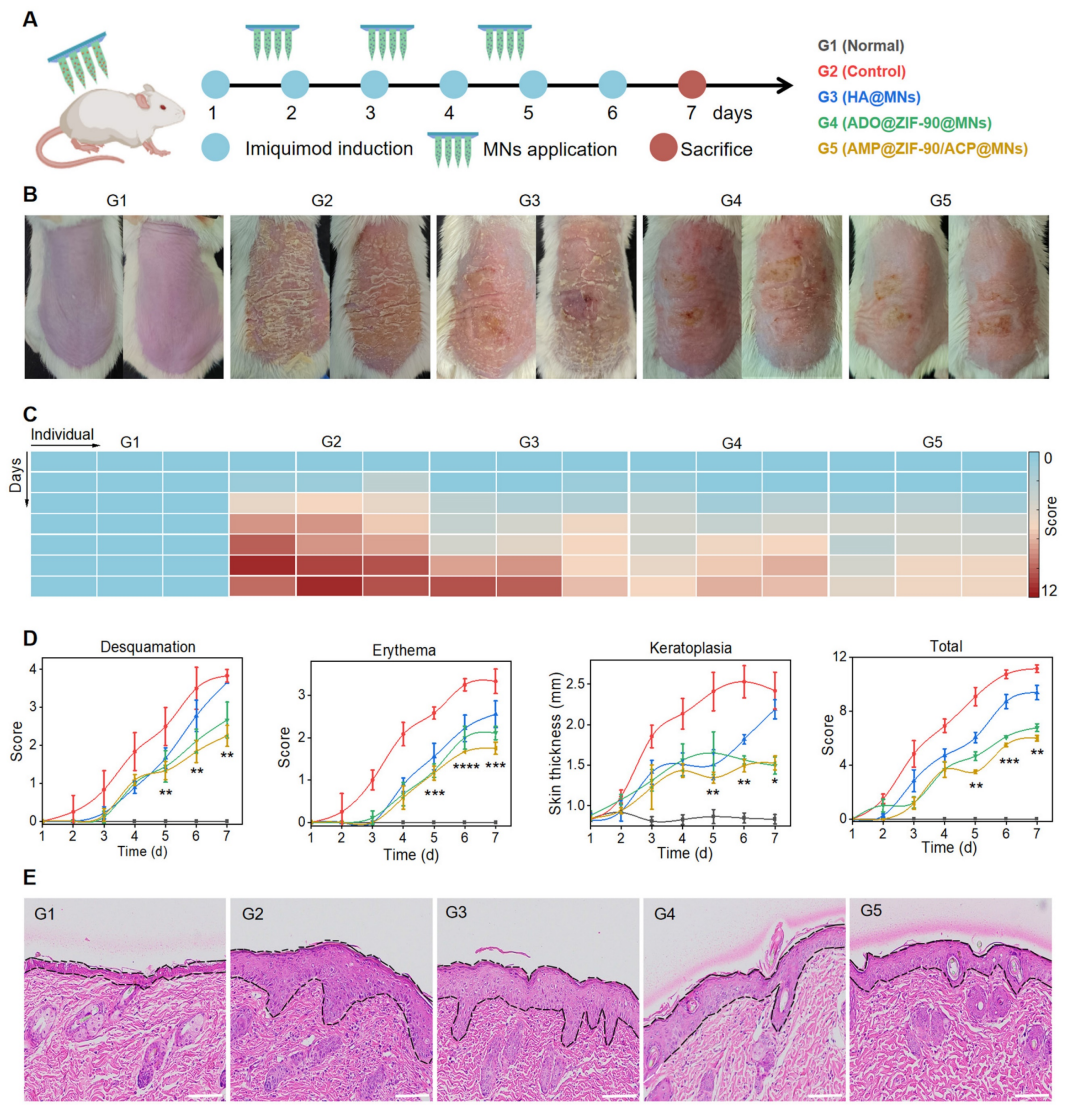
Evaluation of the anti-psoriasis effect. (A) Schematic diagram of experimental schedule. (B) Representative photographs of mice dorsal skin after different treatments. (C) Heatmap of total PASI score of various groups. (D) PASI evaluation of desquamation, erythema, keratoplasia, and total score of different groups during the treatment period (mean± S.D,* n* = 4). (E) The corresponding H&E staining section of psoriasis-like skin lesions after various treatments. All scale bars: 100 μm. (* *P* < 0.05, ** *P* < 0.01, *** *P* < 0.001, **** *P* < 0.0001 *versus* the control group.

The fabrication procedure of AMP@ZIF-90/ACP@MNs is depicted in **Figure 2A**. The accumulation of AMP@ZIF-90/ACP to the needle tips was achieved through a multiple centrifugation micro molding technology, resulting in a notable darkening of the needle tips in comparison to the bottom (**Figure 2B-C**). This is advantageous for precise delivery of drugs to lesion sites. Each MN patch comprised 144 needles (12 × 12) with a length of 1200 μm. The quadrangular pyramid-shaped needles, which retained their structural integrity, were arranged in a uniform manner on the base substrate, as observed from both the lateral (**Figure 2D**) and the superior (**Figure S7**) perspectives. The pyramid-shaped needles were designed with the intention of facilitating deep penetration, which was indeed a necessary quality for the efficient delivery of drug-loaded nanocrystals into the lesion tissue 44. Furthermore, the 2D CLSM micrograph (**Figure 2E**) and 3D reconstruction image (**Figure 2F**) provided additional confirmation of the accumulated distribution of nanocrystals in the tips of AMP@ZIF-90/ACP@MNs.

### Skin penetration capability and dissolution behavior

It is a prerequisite for transdermal drug delivery that the skin be penetrated by MNs. The force displacement curve exhibited sufficient mechanical strength of AMP@ZIF-90/ACP@MNs (**Figure S8**). As illustrated in **Figure S9**, a uniform array of penetration sites was clearly discernible on the dorsal skin of mice following the administration of MN patch, with a penetration rate exceeding 95%. In addition, histological examination of the insertion site using H&E staining confirmed the formation of microchannels within the skin (**Figure 2G**). To gain further insight into the penetration depth, the puncture sites were examined using CLSM tomography. As illustrated in **Figure 2H**, the fluorescence signal of C6@ZIF-90@MNs reached a depth of 400 μm. In conclusion, the results demonstrated that the MNs patch had the requisite mechanical strength to successfully penetrate the cutaneous barrier.

As the needle matrix material of AMP@ZIF-90/ACP@MNs, hyaluronic acid is well-known for its favorable biodegradability and hydrophilia. Therefore, it was anticipated that the MNs patch would disintegrated rapidly when exposed to skin interstitial fluid. The dissolution behavior of AMP@ZIF-90/ACP@MNs was subsequently evaluated following their penetration into the agarose gel. As illustrated in **Figure 2I**, the needle bodies promptly underwent dissolution and diffusion, resulting in complete detachment from the base within 5 min. In summary, the proposed dissolving MN patch was qualified to be an effective technique for embedding nanocrystals into the skin tissue.

### *In vivo* biodistribution analysis

To assess the duration of nanocrystal accumulation at the lesion site, the fluorescence signal variation in psoriasis-like model mice at different time points was visualized through an *in vivo* imaging system (IVIS). As illustrated in **Figure 2J**, both the subcutaneous injection and MNs group exhibited robust fluorescence signals at the lesion site at 0 h. The fluorescence signal persisted at the lesion site of the MNs after 24 h, whereas that of the subcutaneous injection group was markedly diminished at 12 h and undetectable at 24 h. Quantitative analysis (**Figure 2L**) corroborated that MNs administration facilitated prolonged accumulation at the administration site in comparison to subcutaneous injection. To gain further insight into the biodistribution dynamics, the major organs (heart, liver, spleen, lung, and kidney) and lymph nodes were examined 24 h post-administration. As illustrated in **Figure 2K**, no fluorescence signal was discerned following MN administration, whereas a pronounced fluorescence signal was observed in the liver subsequent to subcutaneous injection. These results demonstrate that MNs can facilitate a stable “zone accumulation” strategy, which confines the drug to the superficial lesion site over an extended period. This is due to the fact that MNs can generate hundreds of microchannels at the lesion tissue and uniformly deposit nanocrystals, thereby creating multiple *in situ* drug reservoirs. This further substantiates the notion that a MNs-based regional delivery system is an optimal approach for effectively treating dermatological diseases 45.

### Evaluation of the anti-psoriasis efficacy

The efficacy of various treatment modalities was assessed in a murine model of psoriasis-like dermatitis, induced by the topical application of imiquimod cream to the dorsal skin. The experimental schedule is illustrated in **Figure 3A**. The mice were randomly divided into five groups: G1: Normal mice, G2: Model mice without treatment (Control), G3: Blank MNs (HA@MNs), G4: ADO loaded ZIF-90 MNs (ADO@ZIF-90@MNs), and G5: AMP and ACP co-loaded ZIF-90 MNs (AMP@ZIF-90/ACP@MNs). As illustrated in **Figure 3B**, the dorsal skin of the model mice exhibited distinctive characteristics of psoriasis, including the presence of silvery white scales, erythema, and skin thickening, in comparison to the normal group. The ADO@ZIF-90@MNs group demonstrated a pronounced inhibitory impact on psoriatic dermatitis, substantiating the efficacy of ADO in psoriasis therapy. In contrast, the AMP@ZIF-90/ACP@MNs group exhibited the most pronounced alleviation of symptoms, which may be attributed to the sustained production of ADO under the *in situ* catalytic reaction. Besides, the Psoriasis Area and Severity Index (PASI) score was utilized as a key indicator to quantify the severity of psoriasis. As illustrated in **Figure 3C-D**, the PASI score and heatmap were alignment with the findings visualized from the photographs, indicating that the AMP@ZIF-90/ACP@MNs group exhibited a more pronounced overall symptom relief. Histological examination of the skin lesions was conducted to ascertain the thickness of the epidermis (**Figure 3E**). While all treatment groups demonstrated varying degrees of deceleration in psoriasis progression, the AMP@ZIF-90/ACP@MNs group exhibited the most pronounced efficacy, with an epidermal thickness approximately 2.3 times thinner than that of the control group. These findings substantiate the viability of the intelligent *in situ* catalysis approach in psoriasis management. It is noteworthy that the blank HA@MNs also demonstrated a degree of efficacy. This may be explained by the fact that the mechanical stimulation generated by MNs penetration provides a multidimensional stimulation signal that motivates the skin's self-healing system. This improves the microcycle, promotes skin metabolism, and benefits the normalisation of cell function of keratinocytes, thus ameliorating the symptoms of psoriasis 46-48.

### The mechanism of immunomodulatory effect

**Figure 4:**
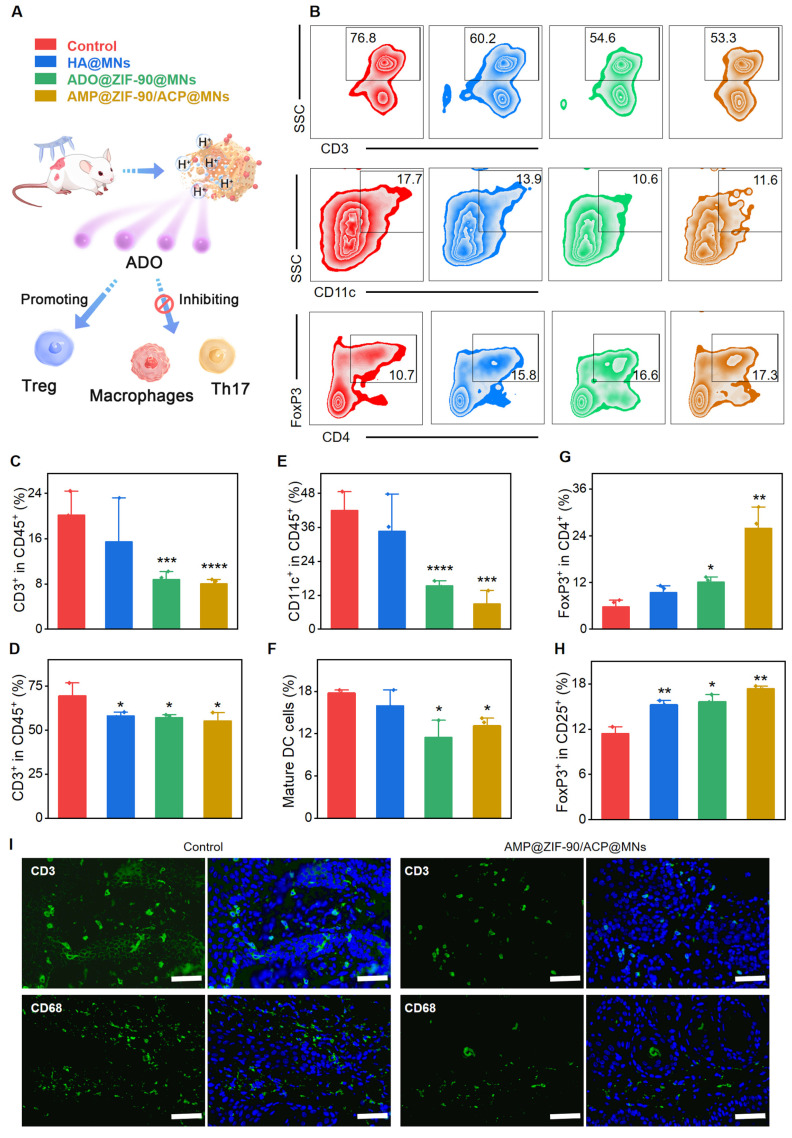
Evaluation of the immunosuppressive mechanism. (A) Schematic diagram of the immunosuppressive mechanism of ADO. (B) Representative flow cytometry charts of mice lymph nodes. Quantification of T cells in (C) dorsal skin lesion and (D) lymph nodes after various treatments. Quantification of mature DCs in (E) dorsal skin lesion and (F) lymph nodes after various treatments. Quantification of Treg cells in (G) dorsal skin lesion and (H) lymph nodes after various treatments. (I) Immunofluorescence staining of CD3 and CD68 in dorsal skin lesion sections of control group and AMP@ZIF-90/ACP@MNs group. (mean ± S.D, *n* = 3). All scale bar: 50 μm. (*P < 0.05, **P < 0.01, ***P < 0.001, ****P < 0.0001* versus* the control group.

To elucidate the immunomodulatory effects, the evolution of the immune population was characterized before and after various treatments. Specifically, the lymph nodes and psoriatic skin were subjected to dissection and subsequent analysis via flow cytometry. As revealed in **Figure 4A-F**, a remarkable reduction in the T cell (CD45^+^CD3^+^) population was observed in the psoriatic skin of the AMP@ZIF-90/ACP@MNs group (55.3) and ADO@ZIF-90@MNs group (57.1), while the T cell population in the psoriasis control group was 69.5. Additionally, a slight decrease in T cells was observed in lymph nodes after treatment with different MNs, indicating an overall suppression of the immune system.

DCs play a pivotal role in T cell activation, and the degree of DC activation is positively correlated with the severity of psoriasis. It is noteworthy that the percentage of DCs (CD11c^+^) in the psoriasis-like skin lesion of the control group was as high as 41.9, while that of the AMP@ZIF-90/ACP@MNs group and ADO@ZIF-90@MNs group was reduced to 8.9 and 15.4, respectively. With regard to the lymph nodes, the percentage of matured DCs (CD80^+^CD86^+^) in the AMP@ZIF-90/ACP@MNs group and ADO@ZIF-90@MNs group was markedly lower than that of the control group, thereby indicating a considerable immunosuppressive effect exerted by ADO. It is noteworthy that the control group and the HA@MNs group exhibited a larger error in T cell (CD45^+^CD3^+^) and DCs (CD11c^+^) count compared to the other two groups. This discrepancy is likely due to the individual animal differences inherent to the experimental design. These results were corroborated by immunofluorescence staining, which revealed a reduction in the infiltration of inflammatory T cells (CD3^+^) and macrophages (CD68^+^) in the skin lesion following treatment with AMP@ZIF-90/ACP@MNs (**Figure 4I, Figure S10**).

Regulatory T (Treg) cells are capable of exploiting their functional adaptability to inhibit the activation and proliferation of immune effector cells. This in turn allows for the restoration of immunological self-tolerance and homeostasis. Numerical and functional defects of Treg cells have been identified as a potential mechanism in the pathogenesis of psoriasis 49. Consequently, we proceeded to quantity the infiltration of Treg cells in both the skin lesions and the lymph nodes. As illustrated in **Figure 4G-H**, the mean percentage of FoxP3^+^ Treg in the skin lesion of the AMP@ZIF-90/ACP@MNs group (26.0) was markedly higher than that of the other groups (5.74 and 12.13 for the control group and ADO@ZIF-90@MNs group, respectively). Furthermore, the FoxP3^+^ Treg population in the lymph node was found to be significantly elevated following treatment with AMP@ZIF-90/ACP@MNs. Thse findings indicate that ADO exerts immunosuppressive effects by modulating Treg cell metabolism. Collectively, the aforementioned results suggest that the enhanced therapeutic efficacy of AMP@ZIF-90/ACP@MNs is attributed to the sustained immunomodulatory supply, which facilitates the reshaping of the inflammatory ecosystem in psoriatic mice through the regulation and manipulation of local and systemic immune cells.

ADO has the capacity to act as an immunomodulator, thereby mediating the immune homeostasis when exposed to an excessively activated adaptive immune system. Subsequently, we conducted a comprehensive investigation into the mechanisms responsible for the efficacy of MN patch in treating psoriasis. In particular, the IL-23/Th17 axis has been identified as a pivotal pathway in the pathogenesis of psoriasis 50. IL-23 is a crucial initial cytokine in the pathogenesis of autoimmunity. During the pathogenesis of psoriasis, activated dermal DCs secrete IL-23, which induces the differentiation of naive T cells into inflammatory T cells, such as Th17 cells. The excessive proliferation of Th17 cells facilitates the secretion of the pro-inflammatory cytokines IL-17, IL-22, and TNF-α 51. These mediators promote the uncontrollable hyperproliferation of keratinocytes and simultaneously induce the massive infiltration of immune cells into lesional skin 52. In addition, IL-23 can directly suppress the function of Treg cells, thereby further exacerbating the psoriasis syndrome.

**Figure 5:**
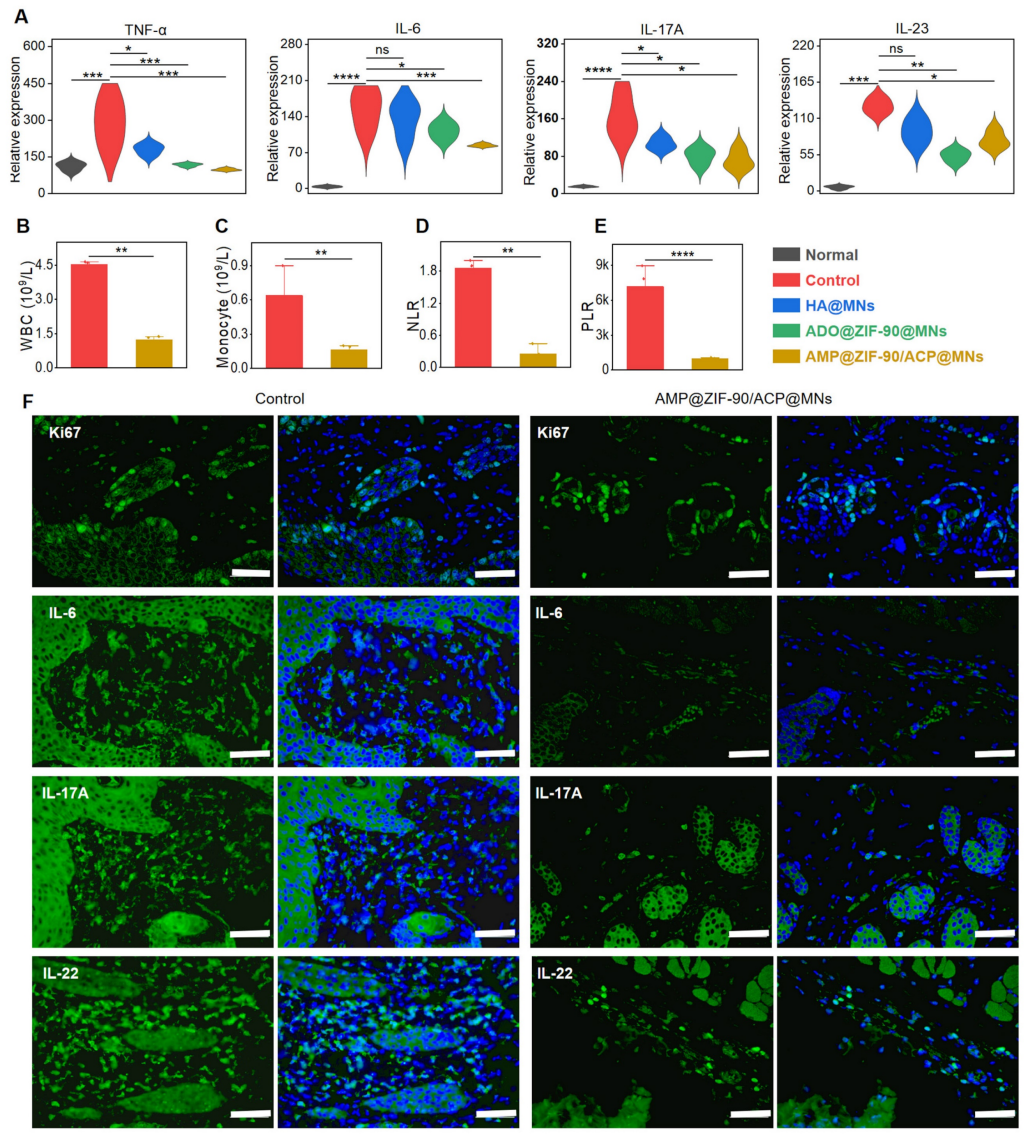
*In vivo* systemic inflammatory response and immunofluorescence staining. (A) Relative expression level of cytokines (TNF-α, IL-6, IL-17A, and IL-23) in the serum after different treatments. Concentrations of (B) white blood cells (WBC), (C) monocytes, (D) neutrophil/lymphocyte ratio (NLR), and (E) platelet/lymphocyte ratio (PLR) in the blood biomarker analysis of the control group and AMP@ZIF-90/ACP@MNs group. (F) Immunofluorescence staining of Ki67, IL-6, IL-17A, and IL-22 in mice dorsal skin lesion of control group and AMP@ZIF-90/ACP@MNs group. (mean ± S.D, *n* = 4). All scale bar: 50 μm. *P < 0.05, **P < 0.01, *** *P*< 0.001, ****P < 0.0001, ns notes not significant *versus* the control group.

To assess the extent of systemic inflammation, the degree of splenomegaly was quantified by calculating the spleen index. As illustrated in **Figure S11A-B**, the spleen index of the control group was 3.7-fold higher than that of the normal group, indicating the presence of severe systemic inflammation. The AMP@ZIF-90/ACP@MNs group exhibited the lowest spleen index among all treatment groups, which was comparable to that of the normal group. The systemic anti-inflammatory effect was also confirmed by measuring the expression level of corresponding cytokines in serum. As illustrated in **Figure 5A**, a notable increase in the secretion of pro-inflammatory factors, including TNF-α, IL-6, IL-17A, and IL-23, was observed during the deterioration of psoriasis. Conversely, a significant decline in these factors was evident following the administration of AMP@ZIF-90/ACP@MNs. The anti-inflammatory effect was additionally corroborated through an examination of the principal blood biomarkers. As illustrated in **Figure 5B-C**, the elevated levels of white blood cells (WBC) and monocytes, which are indicative of abnormal immune function, were significantly reduced in the AMP@ZIF-90/ACP@MNs group. As elevated neutrophils in psoriasis can amplify the inflammatory response by activating platelets, the neutrophil/lymphocyte ratio (NLR) and platelet/lymphocyte ratio (PLR) can be employed to verify the dysfunction of innate immunity arising from psoriasis 53, 54. As illustrated in **Figure 5D-E**, the markedly reduced NLR and PLR values in the AMP@ZIF-90/ACP@MNs group substantiate its exceptional efficacy in psoriasis alleviation. To elucidate the local cytokine milieu, the skin lesions were subjected to further investigation via immunofluorescence staining for IL-6, IL-17A, and IL-22. The results demonstrated a notable reduction in the expression of these inflammatory mediators following treatment with AMP@ZIF-90/ACP@MNs (**Figure 5F, Figure S12**). In addition, Ki67 staining was performed as a conventional marker of aberrant keratinocyte proliferation. In comparison to the model control group, the Ki67 expression in the psoriatic skin was markedly diminished following treatment with AMP@ZIF-90/ACP@MNs, which was in accordance with the results of epidermal thickness measurement. These results demonstrated that the *in situ* catalytic product ADO could effectively decelerate the progression of psoriasis by inhibiting the proliferation of keratinocytes and ameliorating the inflammatory syndrome.

### *In vivo* safety evaluation

The transdermal delivery of drugs via MNs was identified as a minimally invasive and highly safe method. The recovery process of the microchannels created by MN penetration was subsequently investigated following the removal of the base part. As illustrated in **Figure S13**, the microholes in the skin exhibited a gradual reduction and complete recovery after 20 min, indicating that the temporary microchannels could significantly minimize the risk of infection. Additionally, the major organs (heart, liver, spleen, lung, and kidney) were collected and examined via H&E staining. The results demonstrated that no discernible pathological alteration or aberrantion was observed following the administration of diverse treatments (**Figure S14**). The body weights of mice were also recorded throughout the entire treatment period. Despite a slight decline in body weight, which may have been attributable to the modeling of psoriasis (**Figure S15**), no notable discrepancy was observed between the various treatment groups. These results substantiated the superior safety and efficacy of the nanocrystal-integrated MN patch, which could be a promising option for psoriasis management.

## Conclusion

In this study, we successfully constructed a dual pH-responsive metal-organic framework nanocrystal-mediated *in situ* catalytic reaction system, which was further integrated with a dissolving MNs patch for the treatment of psoriasis. The biocompatible AMP@ZIF-90/ACP displayed a high payload capacity and could be enriched in the psoriasis lesion with a long duration after MN patch administration, thereby achieving low-dose administration with high therapeutic efficiency. The long-term hydrolysis behavior of this system resulted in the formation of multiple reservoirs for continuous AMP release and ADO production by dephosphorylation reaction. The *in vivo* experiment results demonstrated that this system had an effective anti-inflammatory effect and a favorable immunomodulatory effect. This system was expected to offer a modular platform for co-delivery of prodrugs and their catabolic enzymes to combat a wide range of diseases.

## Supplementary Material

Supplementary figures.

## Abbreviations

ADO: adenosine
AMP: adenosine monophosphate
ACP: acid phosphatase
DCs: dendritic cells
TNF-α: tumor necrosis factor-alpha
IL-6: interleukin-6
IL-17: interleukin-17
IL-22: interleukin-22
IL-23: interleukin-23
MOFs: metal-organic frameworks
ZIF-90: zeolitic imidazolate framework-90
MNs: microneedle
HA: hyaluronic acid
PVP: Polyvinyl pyrrolidone
DAPI: diamidino-2-phenylindole
EDX: energy dispersive X-ray
SEM: scanning electron microscope
DLS: dynamic light scattering
UV-Vis: ultraviolet and visible spectrophotometer
HPLC: high-performance liquid chromatography
EE: encapsulation efficiency
DL: drug loading
OD: optical density
C6: coumarin 6
MFI: mean fluorescence intensity
SC: subcutaneous injection
SPF: Specific pathogen-free
SD: standard deviation
pXRD: powder X-ray diffraction
CLSM: confocal laser scanning microscope
IVIS: *in vivo* imaging system
PASI: psoriasis area and severity index
WBC: white blood cells
NLR: neutrophil/lymphocyte ratio
PLR: platelet/lymphocyte ratio
