# Supplementary material for: Biomineralized in situ catalytic nanoreactor integrated microneedle patch for on demand immunomodulator supply to combat psoriasis
Source: Theranostics. 2024 Oct 7;14(17):6571–86. doi: 10.7150/thno.101845 (PMC11519795; doi:10.7150/thno.101845)
Supplement: Supplementary file 1 — Supplementary figures. [file thnov14p6571s1.pdf]

Biomineralized in situ catalytic nanoreactor integrated microneedle patch for on demand immunomodulator supply to combat psoriasis

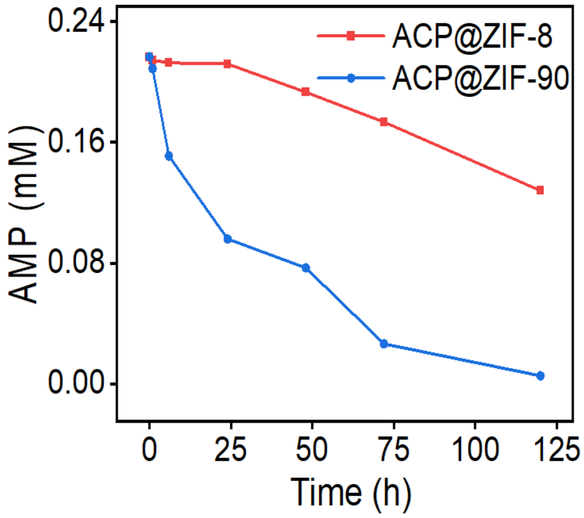

Figure S1 The AMP concentration after immersing in ACP@ZIF-8 and ACP@ZIF-90 suspension, respectively.

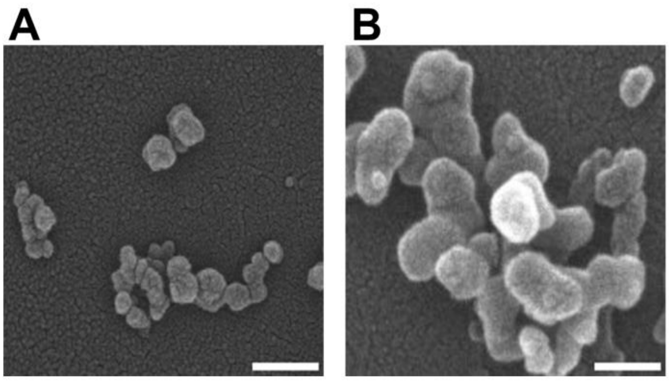

Figure S2 SEM images of (A) AMP@ZIF-90 and (B) AMP@ZIF-90/ACP. Scale bar: 100 nm.

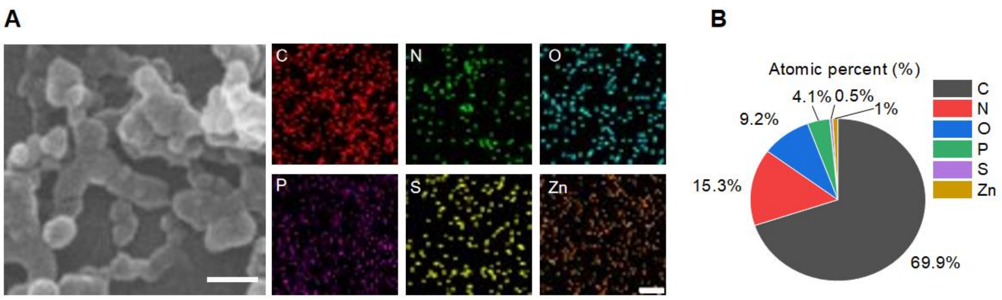

Figure S3 (A) EDX analysis and (B) atomic percent of AMP@ZIF-90/ACP. Scale bar: 100 nm.

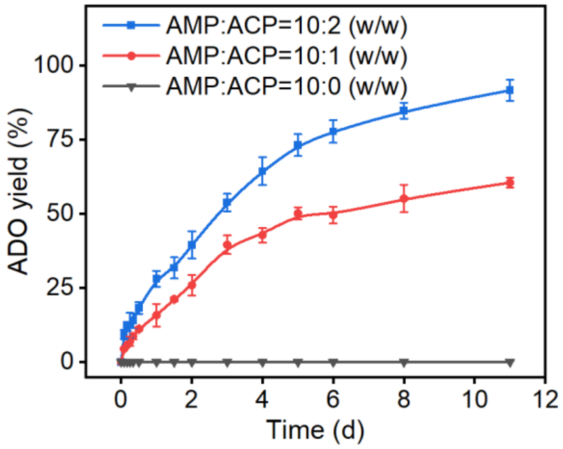

Figure S4 The concentration of generated ADO after immersing AMP@ZIF-90/ACP with different ratio of AMP and ACP in pH 5.5 PBS (mean ± S.D., *n*=3).

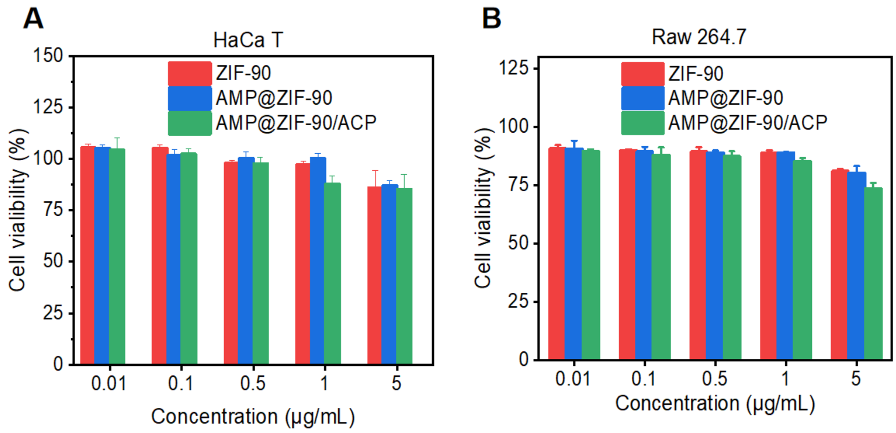

Figure S5 Cell viability of (A) HaCa T and (B) Raw 264.7 cells after incubation with various samples for 24 h (mean ± S.D., *n*=6).

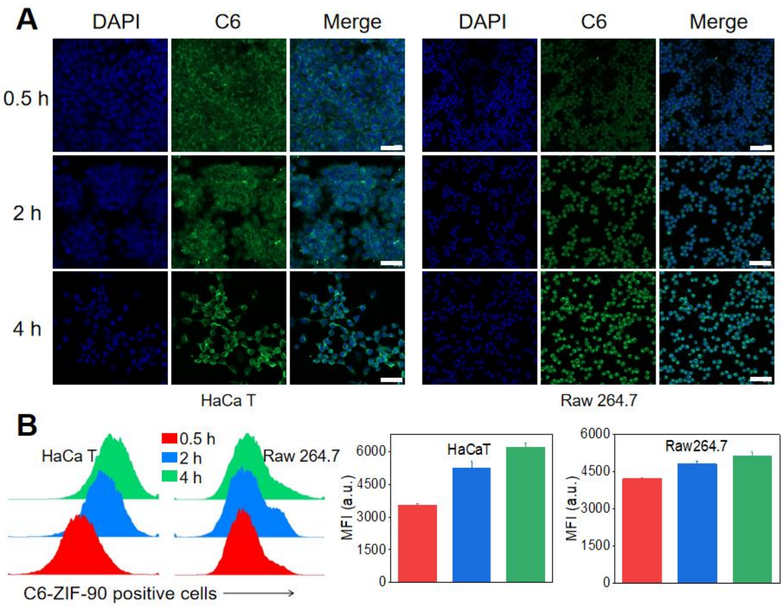

Figure S6 Cellular uptake behavior evaluated by (A) CLSM and (B) flow cytometry, respectively (mean±S.D., n=3). Scale bar: 40 μm.

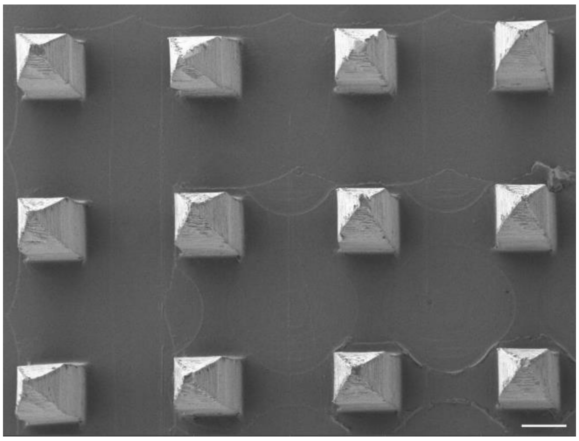

Figure S7 SEM image of AMP@ZIF-90/ACP@MNs patch from top view. Scale bar: 200 μm.

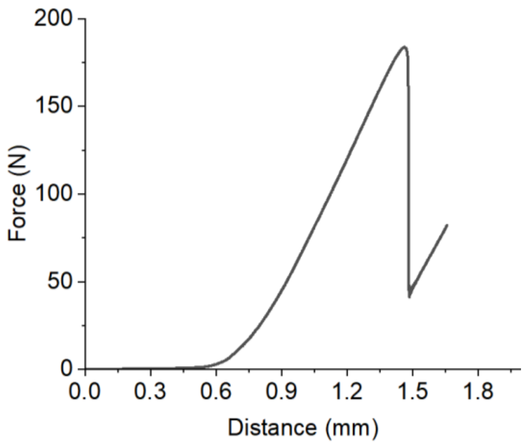

Figure S8 The force displacement curve of AMP@ZIF-90/ACP@MNs.

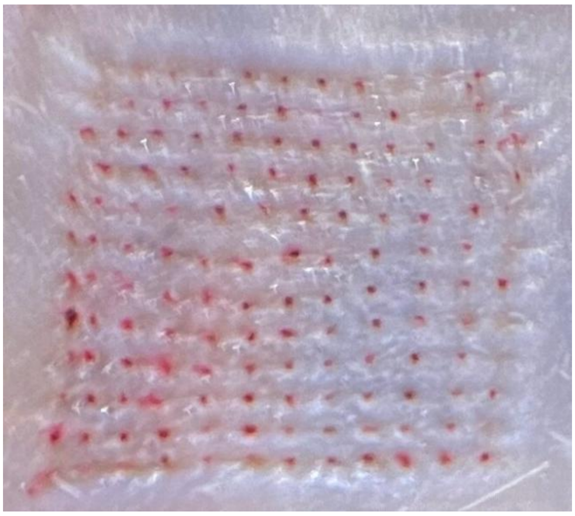

Figure S9 Photograph of mice dorsal skin after administration of C6@ZIF-90@MNs.

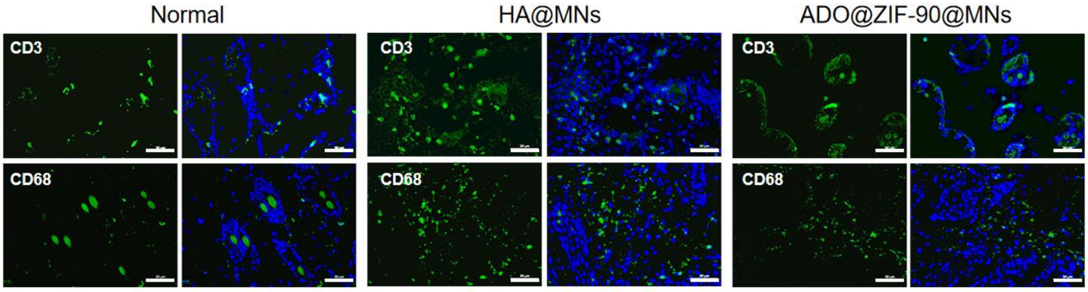

Figure S10 Immunofluorescence staining of CD3 and CD68 in mice dorsal skin lesion sections of Normal, HA@MNs, and ADO@ZIF-90@MNs group. Scale bar: 50 μm.

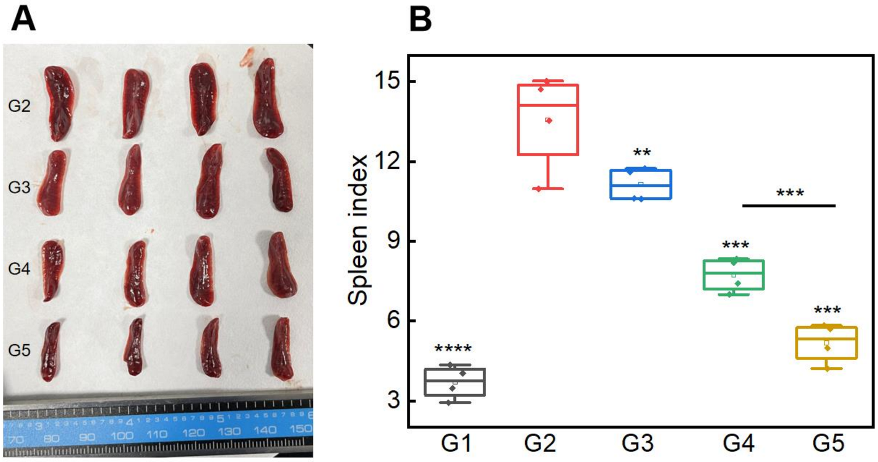

Figure S11 Digital photograph of spleen (A) and (B) spleen index of various groups after treatment (mean±S.D., n=3). (** $P < 0.01$, *** $P < 0.001$, **** $P < 0.0001$ *versus* the control group.

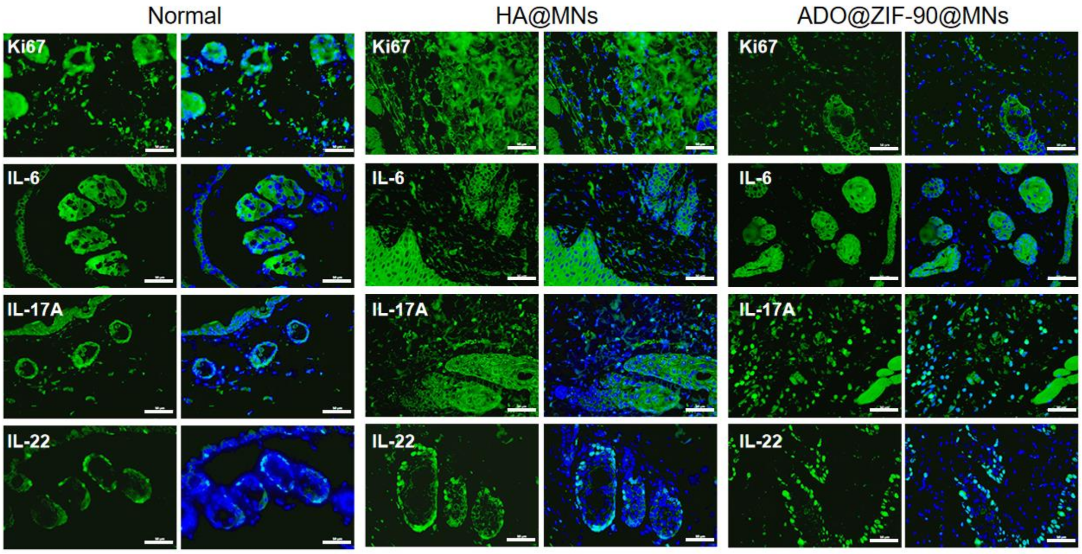

Figure S12 Immunofluorescence staining of Ki67, IL-6 , IL-17A, and IL-22 in mice dorsal skin lesion sections of Normal, HA@MNs, and ADO@ZIF-90@MNs group. Scale bar: 50 μm.

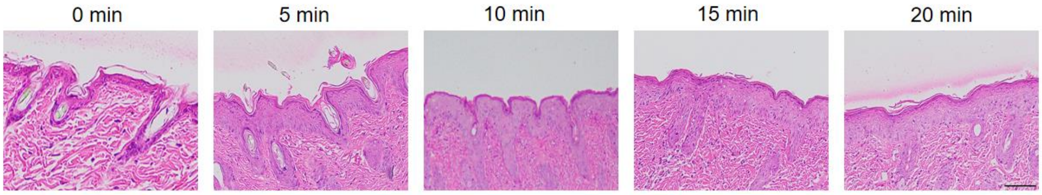

Figure S13 H&E staining of skin after AMP@ZIF-90/ACP@MNs administration at 0, 5, 10, 15, and 20 min. Scale bars: 200 μm.

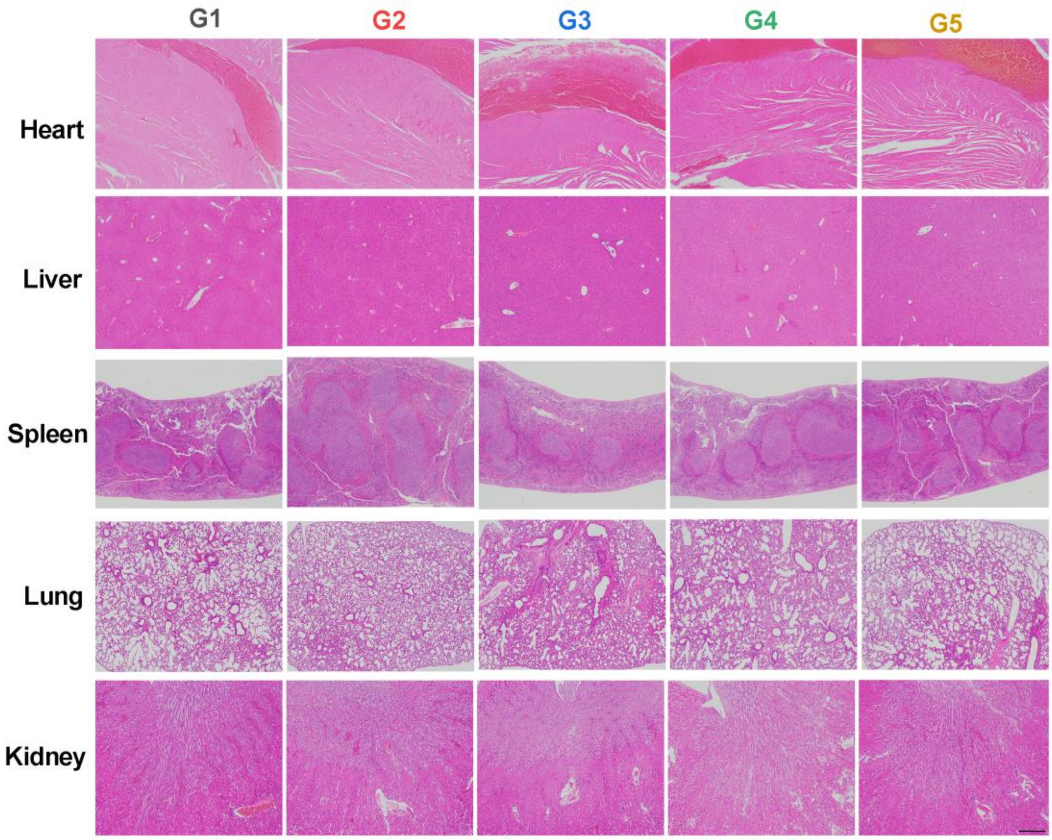

Figure S14 H&E staining of heart, liver, spleen, lung, and kidney of various groups after treatment for histological examination. Scale bars: 200 μm.

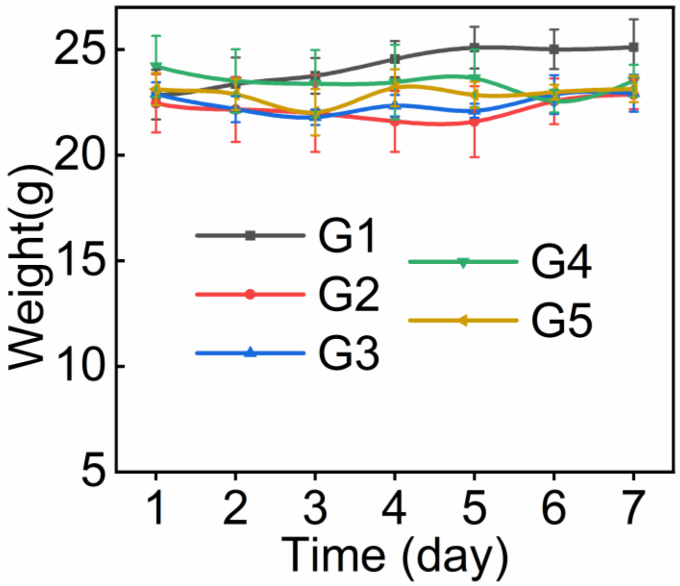

Figure S15 Body weight recorded daily during the period of treatment (mean ± S.D., n=3).
